# Data-driven identification of endophenotypes of Alzheimer’s disease progression: implications for clinical trials and therapeutic interventions

**DOI:** 10.1186/s13195-017-0332-0

**Published:** 2018-01-15

**Authors:** Nophar Geifman, Richard E. Kennedy, Lon S. Schneider, Iain Buchan, Roberta Diaz Brinton

**Affiliations:** 1 Centre for Health Informatics, University of Manchester, Vaughan House, Portsmouth St, Manchester, M13 9GB UK; 2 The Manchester Molecular Pathology Innovation Centre, University of Manchester, Manchester, UK; 3 School of Medicine, University of Alabama at Birmingham, Birmingham, AL USA; 4 Keck School of Medicine, University of Southern California, Los Angeles, CA USA; 5 Leonard Davis School of Gerontology, University of Southern California, Los Angeles, CA USA; 6 Microsoft Research, Cambridge, UK; 7 Department of Pharmacology, College of Medicine, University of Arizona, Tucson, AZ USA; 8 Department of Neurology, College of Medicine, University of Arizona, Tucson, AZ USA; 9 Center for Innovation in Brain Science, University of Arizona, Tucson, AZ USA

**Keywords:** Alzheimer’s disease, Precision medicine, Endophenotypes, Machine learning, Statistical learning, Latent class mixed models

## Abstract

**Background:**

Given the complex and progressive nature of Alzheimer’s disease (AD), a precision medicine approach for diagnosis and treatment requires the identification of patient subgroups with biomedically distinct and actionable phenotype definitions.

**Methods:**

Longitudinal patient-level data for 1160 AD patients receiving placebo or no treatment with a follow-up of up to 18 months were extracted from an integrated clinical trials dataset. We used latent class mixed modelling (LCMM) to identify patient subgroups demonstrating distinct patterns of change over time in disease severity, as measured by the Alzheimer’s Disease Assessment Scale—cognitive subscale score. The optimal number of subgroups (classes) was selected by the model which had the lowest Bayesian Information Criterion. Other patient-level variables were used to define these subgroups’ distinguishing characteristics and to investigate the interactions between patient characteristics and patterns of disease progression.

**Results:**

The LCMM resulted in three distinct subgroups of patients, with 10.3% in Class 1, 76.5% in Class 2 and 13.2% in Class 3. While all classes demonstrated some degree of cognitive decline, each demonstrated a different pattern of change in cognitive scores, potentially reflecting different subtypes of AD patients. Class 1 represents rapid decliners with a steep decline in cognition over time, and who tended to be younger and better educated. Class 2 represents slow decliners, while Class 3 represents severely impaired slow decliners: patients with a similar rate of decline to Class 2 but with worse baseline cognitive scores. Class 2 demonstrated a significantly higher proportion of patients with a history of statins use; Class 3 showed lower levels of blood monocytes and serum calcium, and higher blood glucose levels.

**Conclusions:**

Our results, ‘learned’ from clinical data, indicate the existence of at least three subgroups of Alzheimer’s patients, each demonstrating a different trajectory of disease progression. This hypothesis-generating approach has detected distinct AD subgroups that may prove to be discrete endophenotypes linked to specific aetiologies. These findings could enable stratification within a clinical trial or study context, which may help identify new targets for intervention and guide better care.

**Electronic supplementary material:**

The online version of this article (10.1186/s13195-017-0332-0) contains supplementary material, which is available to authorized users.

## Background

Despite substantial investments in research to find better Alzheimer’s disease (AD) therapies, most drug development efforts end in failure [1–3]; to date, there are no generally effective therapies for AD [1]. Part of the reason for widespread failure of therapeutic development for Alzheimer’s may be due to treating all persons with the disease as if they were the same. One potential route to achieving successful and timely identification of effective therapies is to identify subgroups of patients who may be more responsive to existing and experimental interventions. Given the complexity and progressive nature of AD, there are likely to be distinctive phenotypes and genotypes that respond to candidate therapies differently, and therefore a precision approach to prevention and treatment is critical. Such an approach, where persons with the disease are considered based on an endotype, could identify therapeutics that could delay progression of disease to gain the 5-year window necessary to reduce incidence of the disease.

By employing a data-driven, statistical learning approach, we investigated whether distinct subgroups of AD were apparent in an integrated clinical trial dataset; and whether these subgroups were associated with specific clinical features or existing therapies that might have delayed AD progression. Here, we report that clinically meaningful subgroups can be identified and these might be used to stratify patient populations for better AD management and care.

## Methods

### Study participants and data

Data were derived from an integrated dataset of AD clinical trials and observational studies described previously [4, 5]. The datasets consisted of 18 studies from the Alzheimer’s Disease Cooperative Study (ADCS, http://adcs.org) and the Alzheimer’s Disease Neuroimaging Initiative (ADNI, http://www.adni-info.org) conducted from 1993 to 2012 to analyse the decline in sores on the Alzheimer’s Disease Assessment Scale—cognitive subscale [6] (ADAS-cog), Clinical Dementia Rating—Sum of Boxes [7] (CDR-SB) scale and Mini-Mental State Examination [8] (MMSE) over time. The integrated dataset includes demographics information, cognitive assessments, Apolipoprotein E (ApoE) genotyping, concomitant medication information and blood test data for a total of 4574 participants and 25,164 encounters. All diagnoses of AD were based on National Institute of Neurological and Communicative Disorders and Stroke/Alzheimer’s Disease and Related Disorders Association criteria [9]. Routine use of different medications was captured from the concomitant medication logs using brand and generic names as the search terms. In this study we examined the use of statins, non-statin cholesterol-lowering drugs, AD medications, antidepressants, non-steroid anti-inflammatory drugs (NSAIDs), oestrogens, diabetes medications, vitamin E, omega-3 and derivatives, and medications for long-term asthma management. Medication use was evaluated at the baseline visit of the study into which participants were recruited. A full list of drug groups and search terms is presented in Additional file 1; potential misspelling of drug names was not accounted for. From this dataset we selected those participants with a diagnosis of AD who were treated with placebo or not treated at all, resulting in data from 1160 participants. Of these, 16% of participants originated from the ADNI study while the remaining 84% originated from ADCS studies.

### Latent class analysis

We used latent class mixed modelling (LCMM) with the aim of identifying subgroups of patients with statistically distinct changes in cognitive scores over time, as measured by the ADAS-cog. The observation period for each participant started at beginning of the study they were recruited into and continued for up to 18 months (with a mean ± SD follow-up of 12.8 ± 5.9 months).

We specified linear mixed-effects models with the ADAS-cog as the dependent/outcome variable. Mixed effects were used to account for the likely correlation of repeated measurements within the same participant. We used a linear specification for trajectory shape, and a linear term for time to specify the random effects of the model. The lcmm package in R version 3.2.3 [10] was used to fit the model. We tested the model for 1–10 latent classes and the optimal number of latent classes was assessed using the Bayesian Information Criterion (BIC); the model which had the lowest BIC was selected. At model convergence, a posterior probability of membership of the latent classes was calculated for each participant, who was then assigned exclusively to the class for which the highest probability was obtained. This exclusive class assignment was used in order to allow subsequent characterization of patient subgroups. Other a priori specified patient-level variables, such as medication use and blood test analytes, were used to define these subgroups’ distinguishing characteristics (a full list of these variables is presented in Additional file 2).

### Statistical description of latent classes

Categorical patient characteristics (gender, race, marital status, originating trial/study and ApoE genotype) were contrasted for the latent classes using chi-square tests. Analyses of variance were applied to the continuous variables (age at baseline, baseline ADAS-cog, number of education years and the different blood analytes). Tukey’s method for avoiding type I error was used for post-hoc analysis. Blood analytes examined in this study include those measured in routine biochemistry and haematology tests (Additional file 2). Association of medication use with latent classes was explored with tests of binomial proportions.

To test for differences in the slopes (rate of change) of the ADAS-cog score between subgroups of patients, we used a mixed-effects model implemented in R (using the lme4 package [11]). The model included the group effect, the visit (time) effect and group-by-visit interactions.

All analyses were computed using R version 3.2.3. Results are presented as the main effect with a 95% confidence interval. A significance level of 5% was used for main inferences.

## Results

A total of 1160 AD participants assigned to placebo or untreated arms from nine clinical trials/studies, with at least one assessment on the ADAS-cog (at baseline or thereafter), were included. These involved ADNI [12] and ADCS studies evaluating the effects of simvastatin [13], docosahexaenoic acid supplementation [14], oestrogen replacement therapy [15], B vitamin supplementation [16], rofecoxib or naproxen [17], huperzine A [18], valproate [19] and prednisone [20], selected for their inclusion of a placebo-treated arm and the availability of evaluations for an overlapping duration of follow-up. A total of 4856 observations (ADAS-cog scores) over a period of 18 months were included for our analysis. The number of observations per participant ranged from 1 to 10, with a mean ± SD of 4.2 ± 1.9 ADAS-cog scores per participant.

**Fig. 1:**
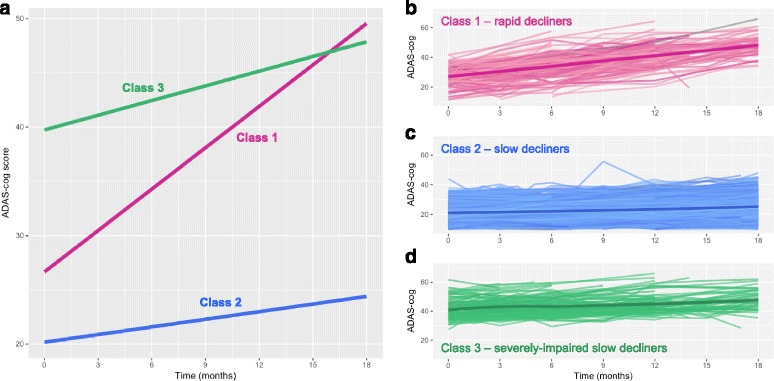
Three classes of ADAS-cog trajectories (higher score is associated with lower cognitive function/greater decline) in participants from placebo or no treatment arms of clinical trials/studies. **a** Disease progression trajectories estimated by our latent class mixed model, where an increase in ADAS-cog scores (*y* axis) indicates worsening of cognitive function. **b**–**d** Individual participant trajectories for each of the three resulting classes (each line represents a single participant). Bold lines represent a smoothing of the data; shaded areas represent the 0.95 confidence interval. ADAS-cog Alzheimer’s Disease Assessment Scale—cognitive subscale

Our analysis of integrated data from these studies identified three subgroups of AD patients displaying unique trajectories of disease: 10.3% in Class 1, 76.5% in Class 2 and 13.2% in Class 3 (with mean posterior probabilities of 0.82, 0.93 and 0.79 respectively). All three classes, on average, exhibited cognitive decline over time. Participants within Class 2 and Class 3 exhibited moderate or slower cognitive decline (with varying starting points) whereas Class 1 demonstrated a steeper decline (Fig. 1).

At baseline, no differences in gender, race, marital status or ApoE genotype were evident between the three classes of cognitive decline (Table 1). In addition, no differences in the rate of decline in cognitive scores were found between participants in placebo-treated arms and those who were not treated at all (the two treatment groups pooled in this dataset).

**Table 1:** Baseline characteristics

	Entire cohort	Class 1	Class 2	Class 3
Number of participants	1160	119	888	153
Duration of follow-up (months)	12.8 ± 5.9	13.6 ± 4.2	13.3 ± 5.7	9.1 ± 6.7
Age	75.6 ± 8.1	73.1 ± 8.9	76.1 ± 7.8	74.7 ± 8.6
Gender				
Female	590 (50.9)	58 (48.7)	443 (49.9)	89 (58.2)
Male	472 (40.7)	50 (42.0)	367 (41.3)	55 (35.9)
Missing	98 (8.4)	11 (9.2)	78 (8.8)	9 (5.9)
Education	14.0 ± 3.2	14.8 ± 3.2	13.9 ± 3.3	14.0 ± 2.9
Race				
Asian	8 (0.7)	1 (0.8)	6 (0.7)	1 (0.7)
African American	59 (5.1)	6 (5)	45 (5.1)	8 (5.2)
White	1007 (86.8)	103 (86.8)	771 (86.8)	133 (86.9)
Other	28 (2.4)	0 (0)	22 (2.5)	6 (3.9)
Missing	58 (5)	9 (7.6)	44 (5)	5 (3.3)
Marital status				
Divorced	70 (6.0)	8 (6.7)	55 (6.2)	7 (4.6)
Married	800 (69.0)	90 (75.6)	603 (67.9)	107 (69.9)
Never married	26 (2.2)	3 (2.5)	22 (2.5)	1 (0.7)
Widowed	243 (20.9)	16 (13.4)	195 (22.0)	32 (20.9)
Missing	21 (1.8)	2 (1.7)	13 (1.5)	6 (3.9)
ApoE4 carriers (%)^a^	63.5	65.7	63.6	62.6
Statin users	311 (26.8)	23 (19.3)	254 (28.4)	34 (22.2)
Baseline ADAS-cog	23.6 ± 9.6	26.5 ± 6.9	20.4 ± 6.7	40.9 ± 6.3

Baseline characteristics of participants in the entire cohort and in each of the resulting latent classes (in the three-class model). Data presented as mean ± standard deviation or *n* (%) unless stated otherwise

*ADAS-cog* Alzheimer’s Disease Assessment Scale—cognitive subscale, *ApoE4* Apolipoprotein E, allele 4

^a^Percent of those participants with relevant information available

**Fig. 2:**
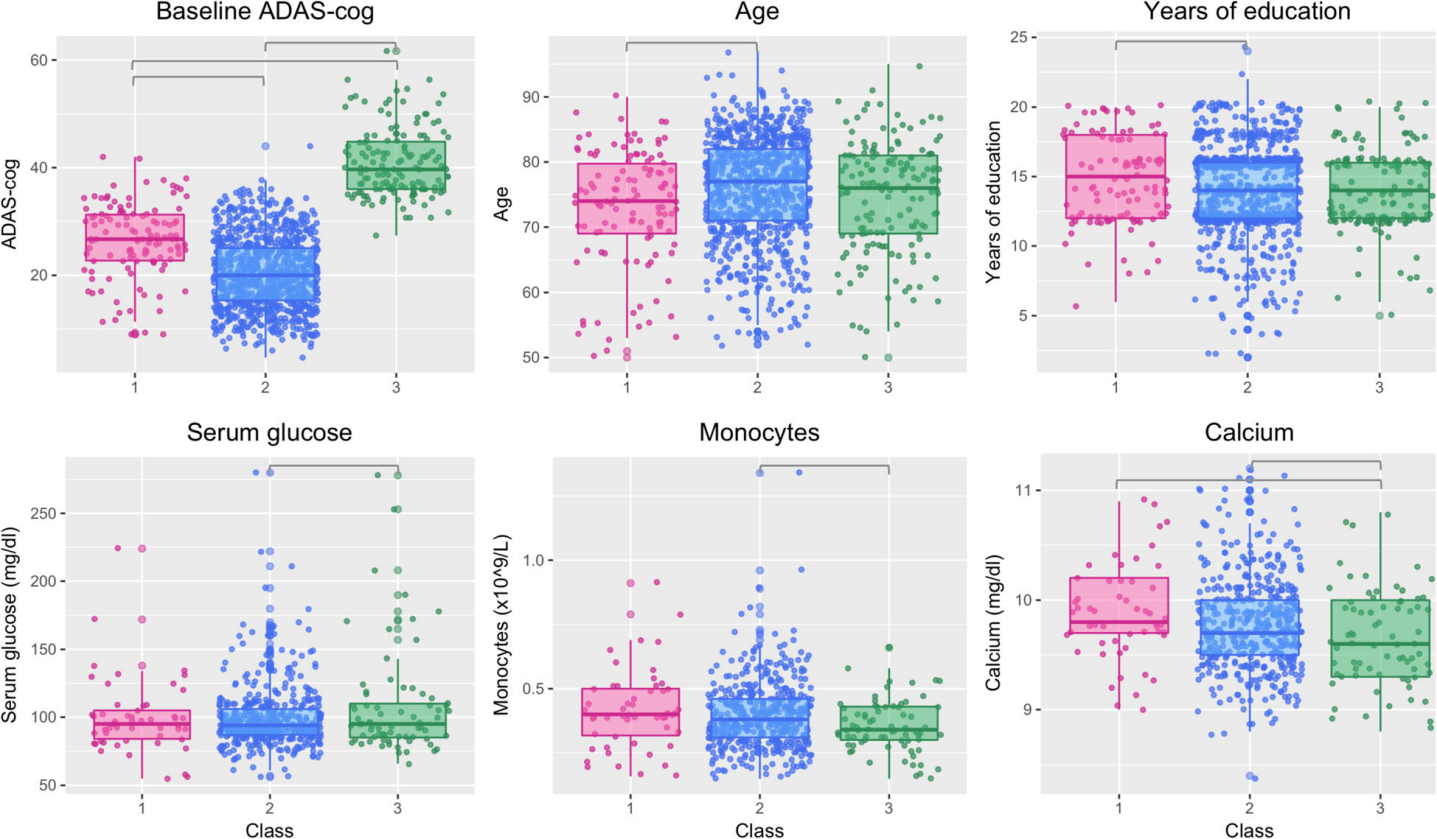
Baseline characteristics that are significantly different between the latent classes (subgroups of patients) identified from clinical trial/study data. Grey lines depict significantly different class pairs (determined by post-hoc analysis). ADAS-cog Alzheimer’s Disease Assessment Scale—cognitive subscale

Participants in Class 1 were overall younger and had a higher mean number of years of education (significantly in comparison to Class 2, *P* < 0.001 and *P* < 0.05, respectively; Fig. 2). Participants in Class 3 demonstrated the highest baseline ADAS-cog scores (*P* < 0.001), lower levels of monocyte counts and calcium levels (significantly in comparison to Class 2, *P* < 0.05) and the highest levels of serum glucose (*P* < 0.05; Fig. 2). Interestingly, Class 2 demonstrated the lowest baseline ADAS-cog scores and a significantly higher proportion of participants with a history of statin use (28.4% vs 19.3% in Class 1 and 22.2% in Class 3, *P* < 0.05). No significant differences between the resulting classes were found for the any of the other evaluated medication groups.

## Discussion

To advance a precision medicine approach to AD, this study aimed to identify clinically relevant endophenotypes of AD, characterized by patterns of disease progression and clinical characteristics, which may prove to be biological endotypes.

We identified subgroups of patients based on a longitudinal analysis of high-quality integrated AD clinical trial data. We considered the possible existence of 1–10 subgroups/classes of disease progression. Using pre-defined criteria, the best fit was identified for a three-class model reflecting three subgroups of disease progression and cognitive decline. Class 1 was characterized by rapid and steep progression of cognitive decline: a mean decline > 10 points in the ADAS-cog. Class 3 was characterized by a greater cognitive deficit at baseline which increased modestly over time, while Class 2 displayed a lower level of cognitive deficit which did not reach the levels seen in Classes 1 and 3.

The three classes had differing clinical features. Class 1 patients tended to be younger and had more education, while those in Class 3 had lower monocyte counts and calcium levels, and higher levels of serum glucose. Age and severity of cognitive impairment have been reported previously as predictors of deterioration [5, 21, 22]. The importance of these characteristics is further demonstrated here, where younger age was found to be associated with a worse trajectory of progression (Class 1) and where lower baseline cognitive impairment was associated with slower decline (Class 2). Higher levels of education, as seen in Class 1, have also been found previously to be associated with risk for rapid cognitive decline [23]. There was no significant difference in gender, race, marital status and ApoE genotype across the three trajectory classes.

The most striking clinical features of the three classes were their pattern of cognitive decline—the structure of interest learned from the data, as well as the proportions of patient membership with each of the classes. The Class 1 rapid decline group had similar prevalence to the severely impaired slow progression phenotype Class 3, but most participants were in the Class 2 phenotype of low baseline cognitive deficit and slow progression of decline thereafter. These findings may have significant impact on powering clinical trials based on predicted magnitude of change over time and potential stratification. It may be the case that future clinical trials may want to consider focusing on recruitment of those patients who would be predicted to fall into Class 1 of rapid decliners as these may increase chances of success. However, the reliability of baseline characteristics in predicating class membership needs to be assessed and validated carefully, potentially in an independent cohort. Furthermore, since Class 1 covers only 10.3% of trial participants, recruiting only those patients would likely encumber trials, as the majority of AD subjects would be excluded. Additionally, to use these trajectories as a prognostic tool, future work should aim to validate our results in a cohort with a significant duration of follow-up, preferably of several years.

The ApoE4 allele is one of the most significant risk factors for sporadic AD [24]. Because the proportion of ApoE4 carriers within each class of disease progression did not significantly differ, ApoE4 genotype is unlikely to drive disease trajectories in the same way that it stratifies populations by the risk of developing AD, at least in mild to moderate AD clinical trial subjects and based on the cognitive measures tested in these. This observation is in agreement with other studies showing that ApoE4 does not significantly influence the rate of cognitive decline in AD [25, 26].

We have previously reported a subset of Alzheimer’s patients on statin therapy demonstrating improved cognitive function [27], supporting the existence of a responder subset of patients who would benefit from treatment with statins. By taking a different approach, these findings are further supported by analysis within the current study, where a subgroup (Class 2: lower ADAS-cog at baseline and slower decline) was also associated with a higher use of statins. Of the different medication types examined in this study, statin use was the only one to be statistically different between the resulting classes. However, it should be noted that this does not necessarily mean that other medications do not have potential therapeutic or preventative effects in AD. For example, it would be very difficult to draw conclusions regarding the effect of NSAIDs as these are not always taken on a regular basis. Unlike statins, diabetes medications, antidepressants or oestrogens, where it is likely that patients prescribed these are taking them on a regular and continuous basis, reported use of NASIDs at baseline might not reflect continuous use of these. Furthermore, here we examined the association of use of different medications with the different patient subgroups and trajectories. This is by no means an in-depth examination of any potential therapeutic effect; it may be the case that the association with statin use is confounded by other factors such as prevalence of cardiovascular comorbidities, differences in prescribing practices or better routine medical care.

A potential limitation of this study stems from the source and pooling of subjects from diverse clinical trials. Firstly, participants recruited into clinical trials may not accurately represent the more general AD patient population; a participant’s reason for joining a trial may differentiate them from the general population, introducing some bias. Secondly, by combining data from different trials, participant variability may be increased since selection criteria for each of these individual trials may have been different. However, since our study utilized data from participants within the placebo and no treatment arms of trials, variability was not influenced by treatment. Furthermore, by including subjects from multiple trials, we are more likely to capture the spectrum of AD patients who may enrol in future trials. Even with potential heterogeneity and variability across the years that studies were conducted, and inclusion and exclusion criteria based on the therapeutic intervention being tested, clinically meaningful subgroups with unique trajectories of disease progression were successfully detected.

This study has generated data-driven, plausible hypotheses about potential endotypes of AD, which could be used to repurpose or seek new targets for treatment or prevention. The unsupervised statistical learning technique used here has also been applied to type 2 diabetes, identifying meaningful patterns of pre-disease and BMI [28]. Similarly, model-based machine learning approaches have been used to discover important endotypes of allergy and asthma [29]. The growing use of unsupervised statistical and machine-learning methods, applied to large-scale patient-level data, shows great promise for better longitudinal characterization of AD. This may in turn lead to better precision diagnostics and more precise interventions. Future work should focus on identifying molecular biomarkers that distinguish between the trajectories identified here. This will provide more precisely defined endotypes which may be used to better stratify patients as well as inform on biological mechanisms driving disease progression.

## Conclusion

We identified three clinical phenotypes of AD, with distinct trajectories of slow decline, severely impaired but slow decline, or rapid decline. Further research is needed to discover the biological mechanisms that may explain these subgroups as endotypes. Additionally, this study demonstrates how precision medicine approaches to AD can be informed by learning from existing datasets such as clinical trials. The findings presented here have the potential to contribute to more effective targeting of trials, medications and other interventions for the benefit of Alzheimer’s patients, thus potentially impacting AD patient management and care significantly.

## Additional files

Additional file 1: Full list of drug groups and search terms used to evaluate medication use at baseline (XLSX 42 kb)

Additional file 2: List of a priori specified patient-level variables at baseline that were used to define the identified classes distinguishing characteristics (XLSX 40 kb)

## Abbreviations

AD: Alzheimer’s disease
ADAS-cog: Alzheimer’s Disease Assessment Scale—cognitive subscale
ADCS: Alzheimer’s Disease Cooperative Study
ADNI: Alzheimer’s Disease Neuroimaging Initiative
ApoE: Apolipoprotein E
ApoE4: Apolipoprotein E, allele 4
BIC: Bayesian Information Criterion
BMI: Body mass index
CDR-SB: Clinical Dementia Rating—Sum of Boxes
LCMM: Latent class mixed models
MMSE: Mini-Mental State Examination
NSAID: non-steroid anti-inflammatory drug
SD: Standard deviation
